# Novel curcumin formulations for the therapy of oral cancer: a comprehensive review of mechanisms, delivery systems, and translational prospects

**DOI:** 10.1007/s44445-026-00214-3

**Published:** 2026-07-17

**Authors:** Sina Seyyedhamzeh, Mohammad Sharif Sharifani, Sevda Sadr Shokriyan, Ayat Jafarova, Meysam Ghanbari Saray, Mohammad Amin Shirmohammadi, Niloufar Shahidan, Amin Jafari, Zeynab Pourtaghi, Negin Ghahremani, Adib Biyari, Amir Hossein Barjasteh, Mohammad Hossein Pourhanifeh

**Affiliations:** 1 Scientific Research Center of Azerbaijan Medical University, Baku, Azerbaijan; 2 School of Paramedical Sciences, Isfahan University of Medical Sciences, Isfahan, Islamic Republic of Iran; 3 Therapeutic Dentistry Department, Azerbaijan Medical University, Baku, Azerbaijan; 4 Student Research Committee, School of Dentistry, Alborz University of Medical Sciences, Karaj, Islamic Republic of Iran; 5 Research Hub Institute, Tehran, Islamic Republic of Iran; 6 Student Research Committee, School of Dentistry, Hamadan University of Medical Sciences, Hamadan, Islamic Republic of Iran; 7 Mashhad University of Medical Sciences, Mashhad, Islamic Republic of Iran; 8 Research Center for Biochemistry and Nutrition in Metabolic Diseases, Institute for Basic Sciences, Kashan University of Medical Sciences, Kashan, Islamic Republic of Iran

**Keywords:** Natural product, Nanoparticle, Liposome, Analogue, Oxidative stress, Curcuminoid, Chemoresistance

## Abstract

Oral cancer, particularly oral squamous cell carcinoma, represents a major global health challenge with high rates of morbidity and mortality. Current treatments often encounter limitations such as toxicity and drug resistance. Curcumin, a natural polyphenol derived from turmeric, has shown significant promise as a therapeutic agent due to its anti-inflammatory, antioxidant, and broad-spectrum anticancer activities. However, its clinical application has been limited by poor solubility, rapid metabolism, and low bioavailability. This review examines the potential of novel curcumin formulations developed to address these challenges in oral cancer treatment. It explores advanced delivery systems including nanoparticle-based carriers, liposomes, niosomes, and hybrid technologies that enhance bioavailability and enable targeted delivery. Additionally, the review discusses synthetic curcumin analogues that offer improved stability and potency. These innovative approaches demonstrate enhanced anticancer effects through pro-apoptotic, anti-proliferative, and anti-angiogenic mechanisms, often exhibiting synergistic activity with conventional therapies. This review aims to synthesize current evidence on the mechanisms and efficacy of these advanced curcumin-based strategies and provide future perspectives on their role as safe and effective options in oral oncology.

## Introduction

Oral squamous cell carcinoma (OSCC), the most prevalent head and neck malignancy, constitutes over 90% of all oral cancers. Histologically, OSCC originates from the squamous epithelium lining the oral cavity (Lissoni et al. 2020). Globally, the disease burden is significant, with more than 350,000 new cases diagnosed annually. Despite this high incidence, prognostic outcomes for OSCC patients have seen little improvement in recent decades (Bray et al. 2018), with a concerning rise in cases among younger populations (Mohideen et al. 2021). The five-year survival rate remains discouragingly low at below 50% (Carreras-Torras and Gay-Escoda 2015), and the standard treatment modalities—surgery, radiotherapy, and chemotherapy—often result in substantial morbidity and a markedly diminished quality of life for survivors (Cicco et al. 2021). The etiology of OSCC is strongly linked to modifiable risk factors, predominantly tobacco use and alcohol consumption, which are collectively implicated in approximately 80% of all cases (Siegel et al. 2016).

Curcumin, a polyphenolic compound extracted from Curcuma longa, is among the bioactive substances sourced from natural products that has been widely studied for its broad pharmacological activities, including anti-inflammatory, antimicrobial, anticancer, and antioxidant impacts (Miller 2001; Fallahi et al. 2021). In normal cells, curcumin inhibits carcinogenesis through its antioxidant mechanisms by neutralizing free radicals and upregulating antioxidant enzymes including glutathione peroxidase (GPx), catalase (CAT), and superoxide dismutase (SOD) along with protein markers like HO-1 and Nrf2. Conversely, in cancer cells, curcumin functions as a pro-oxidant by triggering DNA damage and apoptosis, elevating reactive oxygen species (ROS) levels, and ameliorating the effectiveness of chemotherapy through sensitizing drug-resistant cells. Thus, curcumin exhibits a dual function in cancer, acting as an antioxidant in prevention and as a pro-oxidant with therapeutic advantages (Gupta et al. 2020).

**Fig. 1:**
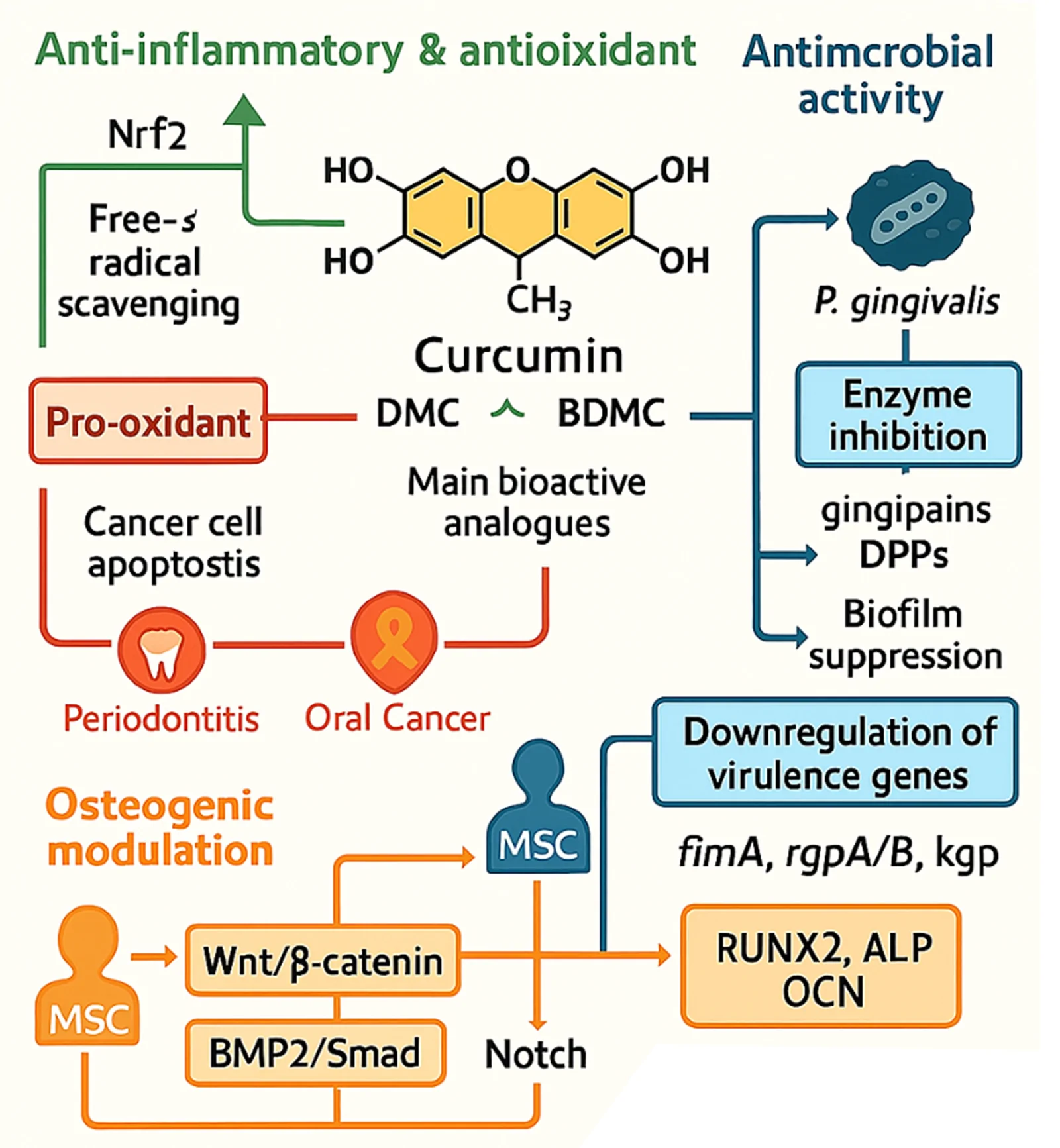
Schematic representation of the therapeutic application of novel formulations of curcumin in the treatment of oral diseases based on cellular mechanisms. The figure was created by the authors using PowerPoint and is an original illustration generated based on information obtained from the cited literature

Curcumin has also been shown to possess biological oroprotective properties in various oral pathological conditions (Fig. 1). In a recent clinical trial, curcumin mouthwash was reported to be as effective as chlorhexidine mouthwash in the therapy of gingivitis, and the herbal mouthwash showed higher potential in decreasing gingival inflammation (Divya Bharathi et al. 2024). In 2025, a systematic review and meta-analysis revealed that various forms of Curcuma longa effectively reduced oral mucositis severity and pain in cancer patients, with curcumin mouthwash considerably lowering mucositis incidence during radiotherapy (Amatto et al. 2025).

Despite substantial preclinical data affirming curcumin’s potentials, its clinical application remains constrained, largely due to its limited water solubility, rapid metabolic degradation, low systemic bioavailability, and instability under physiological conditions (Liu et al. 2018). Pharmacokinetic analyses have revealed that even high oral doses result in subtherapeutic plasma levels, thereby compromising its clinical effectiveness (Carolina Alves et al. 2019). To address this limitation, substantial research has focused on enhancing curcumin’s bioavailability and targeted delivery. For instance, in the case of oral diseases, compared to curcumin, curcumin nanoparticles have been revealed to possess antibiofilm and antimicrobial potentials, with better impacts when associated with blue light. In addition, curcumin and its nanoparticles with and without photoactivation were not cytotoxic to human periodontal ligament fibroblast cells (Tonon et al. 2022). This review article aims to discuss the therapeutic potentials of curcumin and its novel formulations in the management of oral cancer, focusing on underlying mechanisms.

## Materials and methods

This review was conducted to summarize current evidence regarding the therapeutic applications of curcumin and its advanced formulations in oral cancer treatment. A literature search was performed using electronic databases including PubMed, Scopus, and Web of Science. The search strategy included combinations of the following keywords: “curcumin”, “oral cancer”, “oral squamous cell carcinoma”, “nanoparticle”, “liposome”, “noisome”, “solid lipid nanoparticles”, “microemulsion”, “drug delivery system”, “targeted delivery”, “combination therapy”, “curcumin analogues”, “apoptosis”, “autophagy”, “chemosensitization”, and “bioavailability”. Relevant articles published from 2000 to 2026 were considered. Studies investigating the anticancer mechanisms, delivery systems, pharmacological effects, and clinical applications of curcumin in oral cancer were included. In vitro, in vivo, and clinical studies were reviewed, with particular emphasis on recent advances in curcumin formulations. Studies lacking relevance to oral cancer or without sufficient methodological information were excluded.

## Curcumin, a multifunctional natural compound with biological effects

Curcumin, a non-flavonoid polyphenolic molecule derived from the rhizome of Curcuma longa L. (turmeric), is the principal curcuminoid associated with numerous medicinal actions, possessing a chemical formula of C_21_H_20_O_6_ and a molecular weight of 368.37 g/mol (Lai et al. 2024). The active ingredients in turmeric roots are mostly curcumin (77%), then demethoxycurcumin (DMC) (17%), bis-demethoxycurcumin (BDMC) (3%), and cyclocurcumin (3%) (Ajanaku et al. 2022). It has a symmetrical structure that comprises two aromatic rings with o-methoxy phenolic groups connected by a seven-carbon chain that includes an α,β-unsaturated β-diketone moiety. This distinctive planar configuration presents both hydrophobic and weak acidic characteristics, resulting in the predominance of the keto form of curcumin in neutral and acidic solutions, while the enol tautomer prevails in alkaline conditions (Priyadarsini 2014).

Curcumin, as a multifunctional compound, affects various biological targets and has been demonstrated to exhibit anticancer, anti-inflammatory, antioxidant, and antimicrobial properties (Table 1) (Agrawal et al. 2023). This multifunctionality is mediated through its capability to interact with and modulate multiple molecular targets and signaling pathways, including transcription factors (e.g., NF-κB, AP-1, STAT3), enzymes (e.g., COX-2, LOX, MMPs), cytokines (e.g., TNF-α, IL-6, IL-1β), and growth factors (e.g., VEGF, PDGF) (Islam et al. 2024). Its anti-inflammatory characteristics are mostly linked to the activation of NF-κB and reduced production of pro-inflammatory cytokines and enzymes. Additionally, curcumin is a strong antioxidant because it removes free radicals, binds to metal ions, and boosts the function of natural antioxidant enzymes like SOD, CAT, and GPx (Menon and Sudheer 2007). Therefore, the dual role in attenuating inflammation and oxidative stress forms the basis of its protective effects against tissue damage in oral diseases (Curylofo-Zotti et al. 2018). Furthermore, curcumin presents notable antimicrobial activity, which includes antibacterial, antifungal, and antiviral effects. This is possible because it stops biofilm from forming, breaks down microbial cell membranes, and affects microbial enzymes, which helps lower the chance of infections (Moghadamtousi et al. 2014). These properties are particularly relevant in managing oral infections such as periodontitis, oral candidiasis, and dental caries (Forouzanfar et al. 2020). Curcumin’s anticancer potentials are also mediated by modulating pathways involved in cell survival and apoptosis (Zahedi et al. 2023). It has been shown to induce cell cycle arrest, downregulate anti-apoptotic proteins like Bcl-2, and activate caspases, which are essential for programmed cell death (Li et al. 2022). Additionally, inhibition of angiogenesis and metastasis through the suppression of vascular endothelial growth factor (VEGF) and matrix metalloproteinases (MMPs) further underscores its potential in preventing and treating oral cancers (Jayaraman et al. 2024).

Curcumin also have protective effects on various organs, including cardioprotective (Yang et al. 2024), neuroprotective (Genchi et al. 2024), and hepatoprotective effects (Farzaei et al. 2018). Moreover, it has therapeutic roles in several cancers, such as non-small cell lung cancer (Salehi et al. 2020), bladder cancer (Pourhanifeh et al. 2021) and cervical cancer (Ghasemi et al. 2019). Despite its therapeutic potentials, the clinical application of curcumin has been limited due to its poor bioavailability, rapid metabolism, and low solubility in water (Memarzia et al. 2021). These challenges encouraged scientists to synthesize new formulations and develop novel drug delivery systems to improve pharmacokinetic profiles of curcumin and translate its therapeutic effects for oral diseases.

## The effect of curcumin on oral cancer treatment: an update on recent studies

Oral cancer ranks as the sixth most common cancer worldwide, with an increasing prevalence observed in young and middle-aged men (Sung et al. 2021). Notwithstanding the progress made in treatment approaches, the prognosis for patients with oral cancer continues to be relatively unfavorable, highlighting the need for the investigation of innovative therapeutic strategies. In recent years, natural compounds have extensively been investigated in the field of cancer therapy (Hosseinzadeh et al. 2025). Among these compounds, preclinical research has demonstrated that curcumin possesses a range of biological activities, including anti-proliferative, anti-inflammatory, and antioxidant potentials, which have been linked to cancer prevention and suppression (Pourhanifeh et al. 2021; Sadri Nahand et al. 2020; Mirzaei et al. 2021; Salehi et al. 2020).

Recent studies have demonstrated that curcumin exhibits differential inhibitory effects on HPV-positive and HPV-negative oral cancer stem cells (CSCs). Specifically, it significantly suppresses cell proliferation, orosphere formation, and miRNA-21 expression in HPV-positive CSCs, indicating its potential as a chemosensitizer in this context (Bano et al. 2018). Furthermore, curcumin modulates critical cellular pathways by inhibiting the activity of transcription factors such as AP-1 and NF-κB, as well as selectively suppressing the transcription of the HPV16/E6 oncogene in HPV-positive oral cancer cells (Mishra et al. 2015). This highlights the therapeutic potential of curcumin for treating high-risk HPV-infected oral cancers.

In addition to its direct effects on CSCs, curcumin’s synergistic potential with other therapeutic agents has been explored. For instance, the combination of curcumin with metformin has shown significant efficacy in inhibiting CSC-driven oral carcinogenesis, reducing tumor volume, and enhancing overall survival in murine models. The combination treatment downregulated CSC markers and inhibited migratory and self-renewal properties of the CSCs, especially in early dysplastic tissues (Siddappa et al. 2017).

In a recent study, the individual and combined impacts of curcumin and cordycepin were assessed on OECM-1 oral cancer cells and normal gingival epithelial cells. Both compounds induced ROS-mediated cytotoxicity, with more pronounced effects on cancer cells than normal cells. Real-time morphological profiling revealed distinct mechanisms: cordycepin primarily exerted cytostatic effects (inhibiting proliferation), whereas curcumin induced cell shrinkage, impaired motility, and inhibited cell division. The combination therapy largely reflected curcumin-driven changes, with cordycepin coexisting without counteracting curcumin’s effects (Voicu Balasea et al. 2026).

Moreover, curcumin has been reported to reduce immunosuppressive states in peripheral blood mononuclear cells (PBMCs) obtained from patients with OSCC. This intervention decreased levels of PD-1 and PD-L1, suggesting its role in enhancing immune responses in the tumor microenvironment (Dash et al. 2025). Curcumin’s apoptotic effects have been compared to those of paclitaxel, revealing a higher specificity for oral cancer cells with minimal impact on normal cells. Curcumin effectively induced apoptosis in tongue squamous cell carcinoma fibroblast cells while showing significantly less cytotoxicity towards normal gingival fibroblasts (Hussein and Khaphi 2023). The combination of curcumin with PARP inhibitors like talazoparib and olaparib has also shown promising results, enhancing apoptotic effects through increased DNA damage and PARP trapping in oral cancer cells. This combination treatment not only deregulated base excision repair (BER) pathways, but also induced cell cycle arrest, underscoring the potential for synergistic therapeutic strategies (Molla et al. 2021). Olaparib has been demonstrated to enhance curcumin-mediated apoptosis by increasing DNA damage through inhibition of the BER cascade (Molla et al. 2020).

In another study, curcumin and the PARP inhibitor veliparib exhibited anti-angiogenic effects by inhibiting NECTIN-4, which plays a crucial role in promoting angiogenesis in oral cancer (Chatterjee et al. 2021). Curcumin effectively inhibits epithelial-mesenchymal transition (EMT) in oral cancer cells by blocking the c-Met signaling pathway, which is essential for cell motility and invasion (Ohnishi et al. 2020). Moreover, curcumin has been shown to reduce the expression of MMP-2 and MMP-9, which are critical for cancer invasion, while also modulating EMT regulators such as Snail and Twist (Lee et al. 2015). The induction of autophagy has also been implicated in curcumin’s anticancer activity, contributing to decreased survival rates of oral cancer cells (Kim et al. 2012). Additionally, curcumin’s inhibitory effects on oral carcinoma cells have been linked to the suppression of Notch-1 and NF-κB signaling pathways, which are pivotal in regulating cell growth and invasion (Liao et al. 2011). Curcumin also promotes the upregulation of insulin-like growth factor binding protein-5 (IGFBP-5) and C/EBPalpha, which are known to suppress head and neck carcinogenesis, further highlighting its multifaceted role in oral cancer treatment (Chang et al. 2010).

In a recent study by Ludwig et al., small extracellular vesicles (sEVs) derived from Jurkat cells were loaded with curcumin via sonication (JCsEV) and evaluated in both in vitro and in vivo models of OSCC. In vitro, JCsEV significantly reduced tumor cell migration, invasion, and metabolic activity compared to free curcumin or unloaded sEVs. In vivo, using the 4-nitroquinoline 1-oxide immunocompetent murine model, intraperitoneal administration of JCsEV for four weeks significantly decreased tumor number and tumor burden, while also preventing body weight loss. This study provides proof-of-concept for sEV-based nanomedicine as a promising therapeutic strategy for OSCC (Ludwig et al. 2026).

Copper supplementation has further amplified the anti-tumor effects of curcumin in oral cancer cells. Increased intracellular copper levels significantly enhanced curcumin’s inhibitory effects on cell viability and migration while inducing oxidative stress and apoptosis (Lee et al. 2016). This combination provides molecular insight into overcoming the insensitivity of oral cancer cells to curcumin treatment, suggesting a new strategy for cancer therapy.

A recent clinical trial has shown that combining curcumin with green tea extract in patients with potentially malignant oral disorders resulted in significant clinical responses and downregulation of molecular biomarkers associated with cancer progression. This emphasizes the potential of utilizing natural dietary agents like curcumin and green tea extract in chemoprevention strategies for oral cancer (Neetha et al. 2020). Table 1 summarizes the recent data on the anti-OSCC potentials of curcumin.

**Table 1:** Recent data on the roles of curcumin and its novel formulations in the treatment of oral cancer

Compound	Dose/concentration	Dosage form	Target (s)	Effect (s)	Model	Ref.
Curcumin	0–50 µM	Free curcumin	miR-21	Anti-proliferative effect against cancer stem cell, downregulation of miR-21 expression	In vitro	(Bano et al. 2018)
Curcumin	100 µM	Free curcumin	NF-κB, AP-1, p53, Bcl-2, Bax, cIAP2	Promotion of tumor suppressor restoration, induction of programmed cell death effects	In vitro	(Mishra et al. 2015)
Curcumin	20 µM	Free curcumin	Sp1, p65, HSF1, NF-κB	Anti-proliferative, transcription factor-modulating, protein expression-reducing, and pathway activity-suppressing effects	In vitro	(Liu et al. 2021)
Curcumin	40 µM	Free curcumin	EGFR, p-EGFR, Akt, ERK1/2, STAT3, MMP-2, MMP-9, uPA, uPAR	Anti-proliferative, cell cycle-arresting, invasion-suppressing, protein expression-reducing, phosphorylation-inhibiting	In vitro	(Zhen et al. 2014)
Curcumin	15 µM	Free curcumin	MMP-2, MMP-9, Snail, Twist, E-cadherin, p53	Anti-proliferative and anti-invasion effect, EMT-repressing	In vitro	(Lee et al. 2015)
Curcumin	15 µM	Free curcumin	c-Met, p-c-Met, ERK, p-ERK, E-cadherin, vimentin, pro-MMP9	Invasion-suppressing, migration-inhibiting, EMT-repressing, phosphorylation-inhibiting, gelatinolytic activity-reducing	In vitro	(Ohnishi et al. 2020)
Curcumin	80 mg/kg	Free curcumin	HO-1	Dysplasia-modulating effects	In vivo	(Maulina et al. 2019)
Curcumin	50, 150 µM	Free curcumin	ROS	Induced cell shrinkage, impaired motility, inhibited division	In vitro	(Voicu Balasea et al. 2026)
Trienone 11	20 µM	Analogue	caspase-3/7/9	ROS- and caspase-mediated apoptotic effect	In vitro	(Utaipan et al. 2020)
WZ37	0.625–40 µM	Analogue	BAD, PTEN, Akt/mTOR signaling	Anti-proliferative effect, pro-apoptotic effect	In vitro	(Zhang et al. 2020)
BDMC-A	10 µM	Analogue	NF-κB, MMP-9, VEGF, STAT3	Anti-invasive, anti-angiogenesis, anti-metastasis, and anti-progression effects	In vitro	(Mohankumar et al. 2021)
FLLL32	16 µM	Analogue	caspase-3/8/9, p38 MAPK signaling; HO-1	Pro-apoptotic effect, anti-tumor activity	In vitro	(Su et al. 2021)
HO-3867	0.25-1 µM	Analogue	caspase-3/8/9, JNK1/2 signaling	Anti-growth and pro-apoptotic effects	In vitro	(Chen et al. 2022)
CLEFMA	1–16 µM	Analogue	p38 MAPK signaling, HO-1	Anti-survival effect; Pro-apoptotic effect; anti-colony formation Anti-tumor growth; pro-apoptotic effect	In vitro *&* in vivo *(Subcutaneous murine SCC-9 xenograft* *Model)*	(Chen et al. 2022)
EF-24	0.25-1 µM	Analogue	MMP-9, NF-κB	Anti-invasive effect	In vitro	(Su et al. 2023)
PAC	1–10 µM	Analogue	Cyclin D1, p21^WAF1^, p53, caspase-3/9	Anti-proliferative; Pro-apoptotic and anti-metastatic effects; redox balance	In vitro	(Semlali et al. 2021)
GO-Y078	0.5-4 µM	Analogue	SMAC/DIABLO (HO)-1	Anti-tumor progression, anti-survival, anti-proliferative, and pro-apoptotic effects	In vitro	(Chien et al. 2022)
Demethoxycurcumin(DMC)	1-100 µM	Analogue	caspase-3/8/9, Bax, Bcl-2, NF-κB	Anti-proliferative, anti-inflammatory, and pro-apoptotic effects	In vitro	(Lee et al. 2022)
MTH-3	I1-20 µM	Analogue	TFEB	Autophagy induction ,Pro-apoptotic effect	In vitro	(Tsai et al. 2022)
L48H37	-	Analogue	caspase-3, cIAP1, XIAP, JNK, p38 MAPK	Reduction of cell viability, sub-G1 phase accumulation, induction of apoptosis	In vitro (SCC-9, HSC-3 cells)	(Manhas et al. 2024)
FM807	In vivo:50–200 mg/kg In vitro: 3.59–25.75 µM	Analogue	Hsp90, EGFR, β-catenin, Cyclin D1, c-Myc, Raf/MEK/ERK, PI3K/AKT	In vitro: Inhibition of cell proliferation, induction of apoptosis, G1 phase arrest In vivo: Suppression of tumor growth, degradation of client proteins	In vitro (CNE1, CNE2 cells) *and in vivo* (CNE1, CNE2 xenografts)	(Ye et al. 2017)

## Novel delivery systems of curcumin in the treatment of oral cancer

**Fig. 2:**
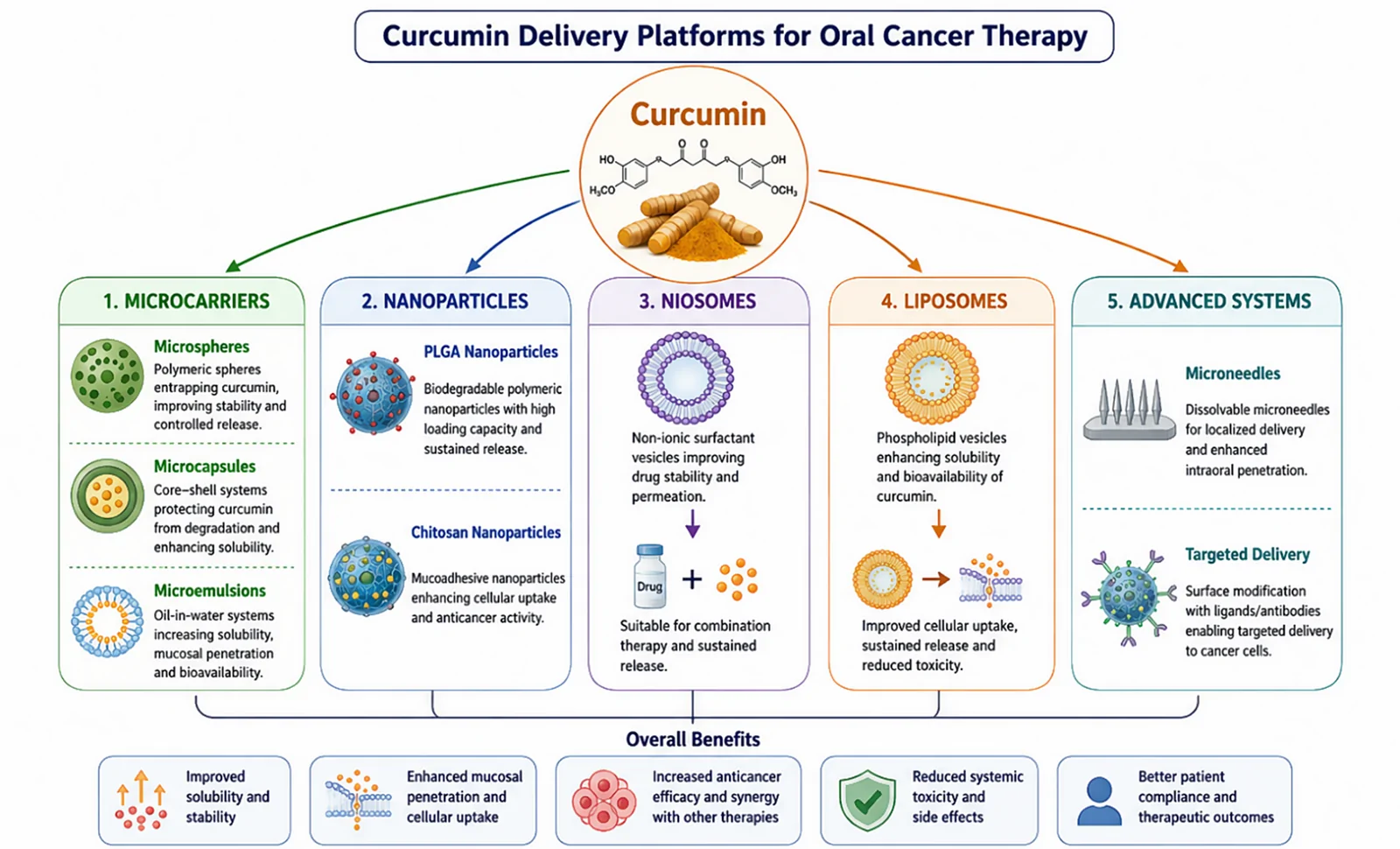
Overview of curcumin delivery platforms for oral cancer therapy, including microcarriers, nanoparticles, niosomes, liposomes, and advanced targeted delivery systems. The figure was created by the authors and does not reproduce previously published illustrations

As mentioned earlier, OSCC, which develops in the oral mucosa, comprises roughly 90% of all oral neoplasms and is frequently linked to unfavorable prognosis and increased mortality rates (Tan et al. 2023). Despite all advances in therapeutic strategies, available treatments are not effective enough. Therefore, novel and cost-effective treatments like natural products are required to avert OSCC progression (Cardona-Mendoza et al. 2022). Notably, curcumin has attracted more attention among these natural compounds for its anticancer potentials. An overview of the major curcumin delivery platforms and their proposed advantages is presented in Fig. 2.

### Challenges with pure form of curcumin

As discussed before, curcumin possesses a broad spectrum anticancer properties against OSCC and head and neck squamous cell carcinoma (Jayaraman et al. 2024, Zhao et al. 2023). However, its therapeutic application faces significant challenges such as poor bioavailability, and low aqueous solubility. The low bioavailability of curcumin is primarily attributed to its rapid metabolism, poor absorption, chemical instability, and rapid systemic clearance (Lopresti 2018). Once curcumin is taken, it undergoes fast metabolism in the liver. It is rapidly transformed into several metabolites, chiefly glucuronides and sulfates, which are subsequently excreted from the body (Nelson et al. 2017). Another notable barrier to curcumin’s efficacy is its limited solubility in water, which varies from around 0.6 µg.mL^− 1^ to 0.011 µg.mL^− 1^ (Zhang et al. 2023). It is estimated to be soluble at 3.216 µg.mL^− 1^ at 25 °C (Priyadarsini 2014). However, multiple approaches have been identified to address these challenges, resulting in an enhancement in curcumin’s therapeutic potential.

### Microcarrier-based delivery systems

Early approaches to improve curcumin delivery were based on microcarrier systems, including microspheres, microcapsules, and microemulsions (Szczęsna et al. 2022; Li et al. 2020). These platforms improve curcumin stability, protect the compound from rapid degradation, and enhance its dispersion in aqueous environments (Pan-On et al. 2022). Microemulsion systems can increase solubility and facilitate mucosal penetration (Yadav et al. 2023), making them attractive for oral applications. Although these strategies have demonstrated improved pharmacokinetic properties compared with free curcumin, their loading capacity, release control, and targeting ability are generally more limited than advanced nanocarrier systems (Nurohman et al. 2026; Wahnou et al. 2025). Therefore, microcarrier technologies may represent an intermediate step in the evolution of curcumin delivery platforms.

### Nanoparticle-based delivery systems

To address the insufficient cellular uptake and limited water solubility of hydrophobic curcumin, it is recommended to encapsulate curcumin in nanoparticles with both positive and negative charges, hence improving its half-life and pharmacokinetics (Khodabux et al. 2021). Nanoparticles ranging from approximately 1 to 100 nm in diameter possess distinctive chemical, physical, and biological properties, rendering them advantageous for drug delivery systems (Biswas et al. 2014). Such characteristics make them superior to existing therapeutic approaches, which exhibit a significant failure rate in radiation therapy for advanced malignancies and the toxicity associated with chemotherapy medicines.

In this regard, in a study, curcumin-loaded chitosan-coated nanoparticles were prepared by using nanoprecipitation technique. Exposure of SCC-9 human oral cancer cells to curcumin-loaded PCL nanoparticles resulted in a significant reduction in cell viability through the induction of apoptosis (Mazzarino et al. 2015). Change et al. designed poly(lactic-co-glycolic acid) (PLGA) nanoparticles loaded with curcumin. In vitro investigation showed that the curcumin-loaded PLGA nanoparticles downregulated Bcl2, increased ROS production, and upregulated caspase-3/9 Bax in CAL27-cisplatin-resistant human oral cancer cells (Chang et al. 2013). Moreover, a solvent-antisolvent precipitation approach was utilized to coat curcumin nanoparticles with 0.5% polyvinylpyrrolidone. Laboratory investigations carried out on the SCC-4 human oral cancer cell line showed anti-tumorigenic and autofluorescent characteristics in a dose-dependent manner. Doxorubicin, a chemotherapeutic drug with a declared necrotic impact, exhibits equivalent cytotoxicity towards human gingival fibroblasts and cancer cells, whereas PVP-stabilized nanocurcumin has a threefold greater selectivity for cancer cells (Essawy et al. 2022).

Notably, nanoparticles are being used for the delivery of anticancer drugs to address chemoresistance in cancer. In a research, 5-fluorouracil (5-FU) and curcumin were combined to develop a nanoemulsion formulation. The augmented anticancer efficacy of this nanohybrid formulation was assessed on SCC152 human hypopharyngeal and SCC090 human tongue cancer cell lines, demonstrating significant apoptosis induction via modulation of Bax, Bcl2, p51, and p53 protein expression (Srivastava et al. 2018). Similarly, Lai and colleagues synthesized γ-polyglutamic acid-coated nanoparticles loaded with gefitinib (Gef) and curcumin, followed by evaluation of its impact on human oral cancer SAS cells and SAS cell xenografted mice models. Free Gef/curcumin had lower anticancer activity than newly formulated nanoparticle. Also, both free Gef/curcumin and γ-PGA-Gef/curcumin nanoparticles triggered apoptotic cell death through mitochondria-dependent, caspase-3, and caspase-9 mechanisms (Lai et al. 2019). A combined treatment of KB 3 − 1 human oral cancer cells with nanocurcumin and cetuximab demonstrated enhanced cytotoxicity. The cytotoxicity was 15.5% for cetuximab alone, 25.4% for nanocurcumin alone, and 46% for their combination. Furthermore, sensitizing KB cells with nanocurcumin prior to cetuximab treatment resulted in even higher cell death compared to both the cetuximab-alone and combination treatment groups, highlighting the potential of this sequential approach (Mukherjee et al. 2022).

Despite the promising anticancer effects reported for nanoparticle-based curcumin delivery systems, several limitations should be considered. Most available studies have been conducted using in vitro models, and therefore the clinical relevance of these findings remains uncertain. Moreover, variations in nanoparticle characteristics, including particle size, surface charge, encapsulation efficiency, and release kinetics, make direct comparison between different formulations challenging. Although nanocarriers may improve curcumin stability and cellular uptake, their long-term safety, biodistribution, and potential toxicity require further investigation through well-designed in vivo and clinical studies.

### Niosomal formulations for synergistic anticancer effects

Niosomes possess a bilayered architecture composed of hydrophilic and hydrophobic parts in an aqueous medium. Since they offer several advantages, including drug stability, improved pharmacokinetics, and reduced adverse effects of the administrated drug, researchers made some endeavors to incorporate curcumin inside them or in conjunction with chemotherapy drug. In this regard, Fazli and his team synthesized curcumin-based niosomes with four concentrations of 4, 8, 16, and 32 µg. Subsequently, human umbilical vein endothelial cells (HUVEC), KB oral cancer cells, and rats were treated with curcumin-loaded niosomes, which exhibited efficacy at a dosage of 16 µg at the cellular level and inhibited the progression of severe dysplasia in rats (Fazli et al. 2022). In a recent investigation, niosomes co-loaded with cisplatin and curcumin were synthesized and assessed. The results showed a continuous and regulated release of both agents. Moreover, the cytotoxic impacts of several formulations on OECM-1 human OSCCs were evaluated, indicating that combination therapy reduced oral cancer cells’ viability in comparison to single treatments (Saberian et al. 2025). In a research conducted by Rezaei et al., niosome nanoparticles were developed to deliver a combination of curcumin and cisplatin for oral cancer treatment. The in vitro release studies demonstrated a controlled and sustained release of curcumin (51%) and cisplatin (48%) over 48 h. Cytotoxicity assays indicated that the combination therapy significantly reduced cell viability of oral cancer cells compared to individual therapies, showcasing a synergistic effect (Rezaei et al. 2024).

### Liposomal carriers for enhanced curcumin bioavailability and sustained release

Liposomes are closed, spherical vesicles containing drugs within the inner aqueous layer. These models, as the second most extensively used vehicle to encapsulate/solubilize curcumin, improve the efficacy and bioavailability of curcumin, and their performance has been enhanced using polymeric conjugates to achieve better therapeutic outcomes. Takahashi et al. prepared two forms of liposome-encapsulated curcumin (LEC) using manufactured lecithins (SLP-WHITE and SLP-PC70). LEC conjugated with SLP-PC70, which had an encapsulation effectiveness of 68% for curcumin, was administered orally to rats to investigate pharmacokinetic parameters. This LEC showed superior curcumin absorption and faster rates, as well as significantly higher plasma concentrations than other delivery methods (Takahashi et al. 2009). Various production techniques, such as ethanol injection, sonication, and thin-layer evaporation, were employed to characterize curcumin liposomes, yielding small unilamellar vesicles (SUVs) and multilamellar vesicles (MLVs). The encapsulation efficiency, size, in vitro release, and cytotoxicity profiles of curcumin liposomes against SCC9 cells varied according to the preparation technique. MLVs demonstrated the greatest encapsulation efficiency; however, they released merely 20% of curcumin over 24 h, enabling a slow and sustained release due to limited drug transfer from the inner lamellae. Conversely, ethanol injection vesicles released up to 80% of the medication, with both SUVs and ethanol injection vesicles demonstrating superior cytotoxicity (IC50 of 5 µM and 2.5 µM, respectively) against SCC9 cells, owing to their reduced size and improved permeability and retention effect. These attributes indicate that smaller liposomes improve curcumin bioavailability, while MLVs are suitable for sustained release applications (Gosangari and Watkin 2012). Moreover, biocompatible polymers such as chitosan have been utilized to enhance the efficiency of conventional liposomal systems, showing promising results regarding bioavailability and pharmacokinetics (Jang et al. 2024).

According to recent evidence, curcumin-loaded liposomal nanoparticles exhibited significantly enhanced anti-proliferative effects in oral cancer cell lines, with a 20% to 30% greater reduction in cell viability compared to free curcumin. The IC50 value for liposomal curcumin was notably lower, indicating superior potency. Drug release assessments showed a sustained release profile over 48 h, suggesting the potential of this nanocarrier system to improve drug solubility, stability, and targeted delivery to cancer cells (Moravedeh et al. 2025). Formulations of cisplatin-curcumin and carboplatin-curcumin nanoliposomes significantly increased cytotoxicity in CAL 27 oral cancer cells compared to control groups. The drug release investigations revealed a sustained release profile, with approximately 22% of cisplatin and 28% of carboplatin released over 52 h, suggesting prolonged therapeutic effects through maintaining drug availability within cancer cells (Saeidi et al. 2024). In 2025, Amiri et al. reported that nanoliposomal formulations of cisplatin and carboplatin with curcumin showed a substantial enhancement in cytotoxic effects on HSC-3 oral cancer cells. The study also revealed a sustained release pattern, with about 21% and 27% of the encapsulated cisplatin and carboplatin released over 35 h, respectively. This controlled release prolongs the therapeutic window and mitigates systemic toxicity in oral cancer therapy (Amiri et al. 2025).

### Safety considerations and current limitations

Although advanced curcumin delivery systems have demonstrated encouraging therapeutic outcomes, several safety concerns remain to be addressed before widespread clinical application. The physicochemical properties of nanocarriers, including particle size, surface charge, and composition, may influence their biodistribution, cellular uptake, and potential toxicity. While most studies have reported acceptable biocompatibility and low toxicity in experimental settings, long-term safety data remain limited. Potential concerns include accumulation within tissues, mucosal irritation following repeated exposure, unexpected immune responses, and variability in biological behavior among different formulations. Furthermore, the majority of available evidence originates from in vitro and animal studies, highlighting the need for comprehensive clinical investigations evaluating long-term safety, tolerability, and pharmacokinetic profiles in patients with oral cancer (Zeng et al. 2022; Shen et al. 2022; Zhang et al. 2022; Hegde et al. 2023).

Currently available delivery systems offer distinct advantages and limitations. Nanoparticles generally provide superior cellular uptake and targeting efficiency, whereas liposomal systems demonstrate favorable biocompatibility and sustained drug release. Niosomes may improve drug stability and facilitate combination therapies, while microcarrier-based systems represent simpler and more economical approaches. Nevertheless, direct comparison among these platforms remains difficult because of differences in experimental models, formulation characteristics, and outcome measures. Therefore, standardized comparative studies are required to identify the most clinically effective delivery strategy for oral cancer treatment (Bautista-Solano et al. 2025, Hussain et al. 2026).

Totally, each of the aforementioned delivery platforms offers distinct advantages and limitations. A comparative summary of these systems, including their key features, loading efficiency, stability, penetration depth, and developmental stage, is presented in Table 2.

**Table 2:** Comparison of curcumin formulations in oral cancer treatment

Delivery System	Advantages	Limitations	Loading Efficiency (%)	Stability	Penetration Depth	Development Stage	Refs.
Nanoparticles (PLGA, PCL, Chitosan)	Enhanced cellular uptake; controlled release; selective cytotoxicity	Limited long-term safety data; mostly in vitro	High (≥ 80%)	Improved in physiological conditions	Cellular penetration	In vitro (SCC-9, CAL27); some in vivo	(Mazzarino et al. 2015, Chang et al. 2013, Lai et al. 2019)
Liposomes (SUVs, MLVs)	High biocompatibility; sustained release; FDA-approved platform	Variable EE (20–80%); stability challenges	20–80%	Moderate; depends on method	Superior for SUVs	In vitro (SCC9, CAL27, HSC-3); PK studies	(Moravedeh et al. 2025, Amiri et al. 2025, Gosangari and Watkin 2012)
Niosomes	Sustained release; reduced adverse effects; synergy with chemotherapy	Limited clinical data; complex synthesis	Moderate to high	Stable in aqueous media	Mucosal penetration	In vitro (OECM-1, KB); In vivo (rat)	(Fazli et al. 2022, Saberian et al. 2025)
Microemulsions / Microcarriers	Improved solubility; easy preparation; mucosal penetration	Lower loading capacity; poor targeting; rapid clearance	Moderate	Limited	Mucosal surface	In vitro; early PK studies	(Zhang et al. 2023)
Curcumin Analogues (e.g., HO-3867, EF24, CLEFMA)	Higher stability; improved potency; selective cytotoxicity	Lack of large-scale clinical trials	N/A	High	Tumor tissue penetration	In vitro; In vivo (xenografts)	(Chen et al. 2022, Chen et al. 2022)
sEV-mediated curcumin	Targeted delivery; reduced tumor burden and number in vivo; improved safety (prevented weight loss)	Mostly preclinical; complex isolation; large-scale production challenges	Moderate (sonication loading)	Stable in physiological conditions	Tissue penetration via sEVs (shown in vivo)	In vitro and in vivo (4-NQO murine model)	(Ludwig et al. 2026)

## Curcumin analogues and derivatives: mechanisms and therapeutic potential in OSCC

In recent decades, multiple modified versions of curcumin, including analogues and derivatives, have been designed to address bioavailability problem. These compounds were developed through incorporation of electronegative groups, introducing of structural asymmetry, and synthesizing of heterocyclic derivatives, exerting improved pharmacokinetic and pharmacodynamic properties compared to curcumin alone (Rodrigues et al. 2021). They have been studied for their potential in treating OSCC both in vivo and in vitro. The anticancer effects of mentioned versions were mediated through decreasing cell proliferation by cell cycle arrest, apoptosis induction and autophagy (Joshi et al. 2023).

The c-Jun N-terminal kinase (JNK) signaling pathway plays a complex and multifaceted role in cancer metastasis, acting as both a promoter and suppressor of tumor spread (Yin et al. 2024). L48H37, a synthetic analogue of curcumin, inhibits the invasive and migratory abilities of nasopharyngeal carcinoma, a type of head and neck cancer, with minimal cytotoxicity. This anticancer impact is attributed to reduced expression and activity of MMP-9, a key mediator in metastasis. Furthermore, L48H37 interferes with 12-O-tetradecanoylphorbol-13-acetate (TPA)-mediated activation of the JNK signaling pathway, and its combination with a JNK antagonist enhances the inhibition of MMP-9 activity and cell migration (Lu et al. 2024). MTH3,a curcumin analogue, upregulates caspase-3, caspase-9, Bax and BAD, and lowers EGFR expression and AKT/mTOR phosphorylation, therefore inducing autophagy in cisplatin-resistant oral cancer cells and activating the intrinsic apoptotic pathway via the Bcl-2 family (Tsai et al. 2022). By suppressing the JAK/STAT3 pathway, converting mutant p53 to wild-type, and inducing both extrinsic and intrinsic apoptosis via JNK1/2 pathways, HO-3867 inhibits OSCC growth in SCC-9 and HSC-3 cell lines as seen by increasing levels of caspase-3, -8, -9, and PARP (Chen et al. 2022). Likewise, by turning off MAPKs and dose-dependent increasing cytochrome C, caspase-3, and caspase-9, EF24 shows greater cytotoxicity than cisplatin, causing apoptosis via the MAPK/ERK pathway (Lin et al. 2017).

Another curcumin analogues, GO-YO78 and FLLL32, upregulate SMAC/DIABLO and HO-1, leading to apoptosis of SCC-9 and HSC-3 cell lines; high HO-1 levels are linked with better prognosis in head and neck cancer patients, implying its therapeutic potential. By means of enhanced HO-1 expression via the p38 pathway, FLLL32 lowers OSCC cell viability by causing apoptosis and G2/M phase cell cycle arrest, hence proving better cytotoxicity than curcumin across several cancer types (Su et al. 2021; Chien et al. 2022). CLEFMA In SCC-9 cells, it causes apoptosis by increasing levels of caspase-3, caspase-8, caspase-9, phosphorylated p38, and HO-1. Oral administration of CLEFMA for 27 days in immunodeficient mice with SCC-9 tumors reduces tumor size and lowers Ki-67 expression, a major proliferation marker, so emphasizing its possible use as an anticancer agent with anti-inflammatory qualities (Chen et al. 2022). Ma et al. focused on the development of a curcumin analogue, AC17, and its delivery via dissolvable microneedles for the treatment of OSCC. This analogue was synthesized by modifying the benzene ring and methylene group of curcumin to enhance its anticancer activity and bioavailability. The AC17-loaded hyaluronic acid microneedle patch (AC17@HAMN) demonstrated superior inhibitory effects on OSCC cells compared to curcumin and other common analogues. Notably, AC17 induced cell cycle arrest and inhibited cell proliferation by activating the FOXO3 pathway. The microneedles exhibited excellent penetration and dissolution properties, allowing for direct delivery of AC17 to tumor tissues, resulting in a significant anti-tumor effect. Furthermore, the AC17@HAMN showed promising biosafety profiles, indicating its potential for clinical applications (Ma et al. 2024).

A recent research carried out by Raouf et al. investigated the anticancer effects of tetrahydrocurcumin (THC), a derivative of curcumin, delivered via phytosomes against oral carcinoma. In vitro tests revealed that THC-phytosomes significantly enhanced anti-proliferative effects on SCC-4 oral cancer cells compared to native THC and cisplatin. THC-phytosomes increased S-phase cell percentages and apoptotic cell populations, while also reducing colony survival and migration rates. The formulation showed higher expression of pro-apoptotic markers and lower oxidative stress, indicating superior efficacy (Raouf et al. 2024). In conclusion, the development of novel formulations of curcumin has demonstrated significant promise in enhancing therapeutic outcomes for oral cancer treatment. Table 1 presents a summary of recent evidence on the therapeutic potentials of various forms of curcumin in the treatment of oral diseases.

## Clinical evidence and translational challenges

Although extensive preclinical studies have demonstrated the anticancer potential of curcumin and its advanced formulations, clinical evidence in oral cancer remains limited. Current human studies have mainly focused on oral health-related conditions, chemoprevention, and treatment-associated complications rather than direct tumor therapy. For example, clinical investigations of curcumin-containing mouthwashes have suggested beneficial effects in reducing oral inflammation and mucositis (Divya Bharathi et al. 2024; Amatto et al. 2025). In addition, preliminary studies combining curcumin with other natural compounds have shown potential chemopreventive effects in patients with potentially malignant oral disorders (Hu et al. 2025). However, limitations including small sample sizes, short follow-up periods, and the absence of large randomized clinical trials restrict definitive conclusions regarding curcumin-based therapies for oral cancer. Future clinical studies should evaluate optimized formulations, appropriate dosing strategies, pharmacokinetic profiles, and long-term safety.

## Future directions

Despite substantial progress in developing curcumin-based therapeutic strategies, several challenges must be addressed to facilitate clinical translation in oral cancer. Future studies should focus on establishing standardized formulations with reproducible physicochemical properties, optimized dosing strategies, and well-defined pharmacokinetic profiles. Comparative investigations between different delivery platforms are required to determine which systems provide the best balance between therapeutic efficacy, stability, safety, and patient compliance. Moreover, large-scale clinical trials are necessary to evaluate the real therapeutic benefit of curcumin formulations in oral cancer patients. Integration of curcumin delivery systems with existing treatment modalities, including chemotherapy, radiotherapy, and immunotherapy, may represent a promising direction for improving treatment outcomes. Advanced approaches such as personalized medicine, targeted delivery, and biomimetic nanocarriers may further enhance the future clinical potential of curcumin in oral oncology.

## Conclusion

Curcumin demonstrates significant therapeutic potential for oral cancer treatment due to its multifaceted ability to combat inflammation, inhibit tumor proliferation, and induce apoptosis. The development of advanced delivery systems, such as liposomal and nanoparticle carriers, has been pivotal in overcoming its poor bioavailability, enabling targeted delivery and enhanced efficacy at tumor sites. Preclinical investigations of these formulations have demonstrated promising antitumor effects, improved bioavailability, and enhanced delivery efficiency. However, clinical evidence supporting their therapeutic application in oral cancer remains limited, and further clinical studies are required to confirm their efficacy and safety. Looking forward, while larger clinical trials are essential to validate optimal dosing, the future of curcumin in oral oncology lies in the continued development and, crucially, the clinical translation of these sophisticated delivery systems to determine whether these promising preclinical findings can be successfully translated into safe and effective clinical applications.

## Data Availability

No datasets were generated or analysed during the current study.

## Declarations

### AI statement

During the preparation of this manuscript, AI-assisted tools were used solely for language editing, grammar correction, and minor textual polishing to improve readability and clarity. No generative AI was used to generate scientific content, data, interpretations, conclusions, or figures. All figures presented in this manuscript were created by the authors. The authors take full responsibility for the accuracy, integrity, and originality of all content, including the figures.

## References

[CR18] Agrawal N, Raza H, Jaiswal M, Lanjhiyana S, Sahoo MK (2023) A Review on the Pharmacological Effects of Curcumin in Diabetes, Cancer, and Neurodegenerative Diseases. Novel Aspects Pharm Res 2 2:59–71

[CR16] Ajanaku CO, Ademosun OT, Atohengbe PO, Ajayi SO, Obafemi YD, Owolabi OA et al (2022) Functional bioactive compounds in ginger, turmeric, and garlic. Front Nutr 9 10.3389/fnut.2022.1012023 PMC9773837 36570131

[CR11] Amatto PPG, Chaves L, França SC, Carvalho JCT, Carmona F, Pereira AMS (2025) Efficacy of different pharmaceutical forms of Curcuma longa or curcumin in reducing oral mucositis severity and incidence in cancer patients: a systematic review and meta-analysis. Front Pharmacol 16:1560729 40242451 10.3389/fphar.2025.1560729 PMC12000115

[CR101] Amiri F, Ghanbarikondori P, Amoozegar H, Kazemi K, Sadrian S, Afshari-BehbahaniZadeh S et al (2025) Synergistic effects of platinum-based drugs and curcumin on liposomal delivery in HSC-3 oral cancer cells. Indian J Clin Biochem 1–7

[CR43] Voicu Balasea B, Stan MS, Radulescu R, Cernega A, Alm K, Musteanu M et al (2026) Differential effects of curcumin and cordycepin on oral squamous cell carcinoma cells: ros-mediated cytotoxicity and real-time morphological analysis. Molecules 31(7) 10.3390/molecules31071221 PMC13074636 41976261

[CR40] Bano N, Yadav M, Das BC (2018) Differential Inhibitory Effects of Curcumin Between HPV+ve and HPV-ve Oral Cancer Stem Cells. Front Oncol 8:412 30319975 10.3389/fonc.2018.00412 PMC6168628

[CR106] Bautista-Solano AA, Dávila-Ortiz G, Perea-Flores MdJ, Martínez-Ayala AL (2025) A comprehensive review of niosomes: composition, structure, formation, characterization, and applications in bioactive molecule delivery systems. Molecules 30(17):3467 40941995 10.3390/molecules30173467 PMC12429857

[CR86] Biswas AK, Islam MR, Choudhury ZS, Mostafa A, Kadir MF (2014) Nanotechnology based approaches in cancer therapeutics. Adv Nat Sci NanoSci NanoTechnol 5(4):043001

[CR2] Bray F, Ferlay J, Soerjomataram I, Siegel RL, Torre LA, Jemal A (2018) Global cancer statistics 2018: GLOBOCAN estimates of incidence and mortality worldwide for 36 cancers in 185 countries. Cancer J Clin 68(6):394–424 10.3322/caac.21492 30207593

[CR74] Cardona-Mendoza A, Olivares-Niño G, Díaz-Báez D, Lafaurie GI, Perdomo SJ (2022) Chemopreventive and Anti-tumor Potential of Natural Products in Oral Cancer. Nutr Cancer 74(3):779–795 34100309 10.1080/01635581.2021.1931698

[CR13] Carolina Alves R, Perosa Fernandes R, Fonseca-Santos B, Damiani Victorelli F, Chorilli M (2019) A Critical Review of the Properties and Analytical Methods for the Determination of Curcumin in Biological and Pharmaceutical Matrices. Crit Rev Anal Chem 49(2):138–149 30252504 10.1080/10408347.2018.1489216

[CR4] Carreras-Torras C, Gay-Escoda C (2015) Techniques for early diagnosis of oral squamous cell carcinoma: systematic review. Medicina oral, patologia oral y cirugia bucal 20(3):e305–e315 10.4317/medoral.20347 PMC4464918 25662554

[CR53] Chang KW, Hung PS, Lin IY, Hou CP, Chen LK, Tsai YM et al (2010) Curcumin upregulates insulin-like growth factor binding protein-5 (IGFBP-5) and C/EBPalpha during oral cancer suppression. Int J Cancer 127(1):9–20 20127863 10.1002/ijc.25220

[CR88] Chang PY, Peng SF, Lee CY, Lu CC, Tsai SC, Shieh TM et al (2013) Curcumin-loaded nanoparticles induce apoptotic cell death through regulation of the function of MDR1 and reactive oxygen species in cisplatin-resistant CAR human oral cancer cells. Int J Oncol 43(4):1141–1150 23917396 10.3892/ijo.2013.2050

[CR48] Chatterjee S, Sinha S, Molla S, Hembram KC, Kundu CN (2021) PARP inhibitor Veliparib (ABT-888) enhances the anti-angiogenic potentiality of Curcumin through deregulation of NECTIN-4 in oral cancer: Role of nitric oxide (NO). Cell Signal 80:109902 33373686 10.1016/j.cellsig.2020.109902

[CR65] Chen PN, Lin CW, Yang SF, Chang YC (2022) CLEFMA induces the apoptosis of Oral squamous carcinoma cells through the regulation of the P38/HO-1 Signalling pathway. Cancers 14(22):551 10.3390/cancers14225519 PMC9688613 36428612

[CR64] Chen CW, Hsieh MJ, Ju PC, Hsieh YH, Su CW, Chen YL et al (2022) Curcumin analog HO-3867 triggers apoptotic pathways through activating JNK1/2 signalling in human oral squamous cell carcinoma cells. J Cell Mol Med 26(8):2273–2284 35191177 10.1111/jcmm.17248 PMC8995445

[CR68] Chien MH, Shih PC, Ding YF, Chen LH, Hsieh FK, Tsai MY et al (2022) Curcumin analog, GO-Y078, induces HO-1 transactivation-mediated apoptotic cell death of oral cancer cells by triggering MAPK pathways and AP-1 DNA-binding activity. Expert Opin Ther Targets 26(4):375–388 35361044 10.1080/14728222.2022.2061349

[CR21] Curylofo-Zotti FA, Elburki MS, Oliveira PA, Cerri PS, Santos LA, Lee HM et al (2018) Differential effects of natural Curcumin and chemically modified curcumin on inflammation and bone resorption in model of experimental periodontitis. Arch Oral Biol 91:42–50 29669267 10.1016/j.archoralbio.2018.04.007

[CR44] Dash P, Nayak S, Parida PK (2025) The Efficacy of Curcumin in Reducing Immunosuppressive States of Peripheral Blood Mononuclear Cells Extracted From Oral Squamous Cell Carcinoma Patients: An In Vitro Study. Cureus 17(1):e77899 39991356 10.7759/cureus.77899 PMC11847154

[CR5] De Cicco D, Tartaro G, Ciardiello F, Fasano M, Rauso R, Fiore F et al (2021) Health-related quality of life in oral cancer patients: scoping review and critical appraisal of investigated determinants. Cancers 13(17):4398 34503208 10.3390/cancers13174398 PMC8431462

[CR10] Divya Bharathi S, Aiswarya SP, Sankar AR (2024) Comparative evaluation of the efficacy of Triphala mouthwash and Curcumin mouthwash in the treatment of gingivitis - A randomized controlled study. J oral biology Craniofac Res 14(4):407–414 10.1016/j.jobcr.2024.05.002 PMC11144734 38832298

[CR89] Essawy MM, Mohamed MM, Raslan HS, Rafik ST, Awaad AK, Ramadan OR (2022) The theranostic potentialities of bioavailable nanocurcumin in oral cancer management. BMC Complement Med Ther 22(1):309 36424593 10.1186/s12906-022-03770-3 PMC9685877

[CR8] Fallahi F, Borran S, Ashrafizadeh M, Zarrabi A, Pourhanifeh MH, Mahabady MK et al (2021) Curcumin and inflammatory bowel diseases: From in vitro studies to clinical trials. Mol Immunol 130:20–30 33348246 10.1016/j.molimm.2020.11.016

[CR29] Farzaei MH, Zobeiri M, Parvizi F, El-Senduny FF, Marmouzi I, Coy-Barrera E et al (2018) Curcumin in liver diseases: a systematic review of the cellular mechanisms of oxidative stress and clinical perspective. Nutrients 10(7):855 29966389 10.3390/nu10070855 PMC6073929

[CR93] Fazli B, Irani S, Bardania H, Moosavi MS, Rohani B (2022) Prophylactic effect of topical (slow-release) and systemic curcumin nano-niosome antioxidant on oral cancer in rat. BMC Complement Med Ther 22(1):109 35440035 10.1186/s12906-022-03590-5 PMC9020014

[CR23] Forouzanfar F, Forouzanfar A, Sathyapalan T, Orafai HM, Sahebkar A (2020) Curcumin for the Management of Periodontal Diseases: A Review. Curr Pharm Design 26(34):4277–4284 10.2174/1381612826666200513112607 32400326

[CR28] Genchi G, Lauria G, Catalano A, Carocci A, Sinicropi MS (2024) Neuroprotective effects of curcumin in neurodegenerative diseases. Foods 13(11):1774 38891002 10.3390/foods13111774 PMC11172163

[CR32] Ghasemi F, Shafiee M, Banikazemi Z, Pourhanifeh MH, Khanbabaei H, Shamshirian A et al (2019) Curcumin inhibits NF-kB and Wnt/β-catenin pathways in cervical cancer cells. Pathol Res Pract 215(10):152556 31358480 10.1016/j.prp.2019.152556

[CR97] Gosangari SL, Watkin KL (2012) Effect of preparation techniques on the properties of curcumin liposomes: characterization of size, release and cytotoxicity on a squamous oral carcinoma cell line. Pharm Dev Technol 17(1):103–109 21091385 10.3109/10837450.2010.522583

[CR9] Gupta N, Verma K, Nalla S, Kulshreshtha A, Lall R, Prasad S (2020) Free radicals as a double-edged Sword: the cancer preventive and therapeutic roles of curcumin. Molecules 25(22) 10.3390/molecules25225390 PMC7698794 33217990

[CR105] Hegde M, Girisa S, BharathwajChetty B, Vishwa R, Kunnumakkara AB (2023) Curcumin formulations for better bioavailability: what we learned from clinical trials thus far? ACS omega 8(12):10713–10746 37008131 10.1021/acsomega.2c07326 PMC10061533

[CR35] Hosseinzadeh M, Bazvand H, Gravand E, Pourhanifeh MH (2025) From inflammation to malignancy: ginsenosides as a bridge between traditional healing and modern therapies for periodontitis and oral cancer. Phytomedicine Plus 100827

[CR124] Hu C, Wang S, Gao Z, Qing M, Tan L, Yang L et al (2025) Curcumin in oral health: mechanisms, clinical evidence, and delivery strategies. Front Pharmacol 16:1661443 41846867 10.3389/fphar.2025.1661443 PMC12989600

[CR107] Hussain S, Arif A, Shah MR (2026) Targeted drug delivery: designing nanocarriers for improved therapeutic action. Chem Commun 10.1039/d5cc07306e 41729230

[CR45] Hussein HA, Khaphi FL (2023) The Apoptotic Activity of Curcumin Against Oral Cancer Cells Without Affecting Normal Cells in Comparison to Paclitaxel Activity. Appl Biochem Biotechnol 195(8):5019–5033 37032374 10.1007/s12010-023-04454-5

[CR19] Islam MR, Rauf A, Akash S, Trisha SI, Nasim AH, Akter M et al (2024) Targeted therapies of curcumin focus on its therapeutic benefits in cancers and human health: Molecular signaling pathway-based approaches and future perspectives. Biomed Pharmacother 170:116034 38141282 10.1016/j.biopha.2023.116034

[CR98] Jang GH, Kim YM, Kim DH, Shin JW, Yoon SY, Bae JW et al (2024) A chitosan/alginate coated nano-liposome to improve intestinal absorption of curcumin for oral administration. Food Sci Biotechnol 33(7):1707–1714 38623436 10.1007/s10068-023-01461-4 PMC11016035

[CR26] Jayaraman S, Veeraraghavan VP, Natarajan SR, Jasmine S (2024) Exploring the therapeutic potential of curcumin in oral squamous cell carcinoma (HSC-3 cells): Molecular insights into hypoxia-mediated angiogenesis. Pathol - Res Pract 254:155130 38277750 10.1016/j.prp.2024.155130

[CR118] Joshi P, Verma K, Kumar Semwal D, Dwivedi J, Sharma S (2023) Mechanism insights of curcumin and its analogues in cancer: An update. Phytother Res 37(12):5435–5463 37649266 10.1002/ptr.7983

[CR85] Khodabux RJ, Parvathi VD, Harikrishnan T, Nanocurcumin (2021) Potential Natural Alkaloid against Oral Squamous Cell Carcinoma. Biomedical Biotechnol Res J (BBRJ) 5(3):252–259

[CR51] Kim JY, Cho TJ, Woo BH, Choi KU, Lee CH, Ryu MH et al (2012) Curcumin-induced autophagy contributes to the decreased survival of oral cancer cells. Arch Oral Biol 57(8):1018–1025 22554995 10.1016/j.archoralbio.2012.04.005

[CR91] Lai KC, Chueh FS, Hsiao YT, Cheng ZY, Lien JC, Liu KC et al (2019) Gefitinib and curcumin-loaded nanoparticles enhance cell apoptosis in human oral cancer SAS cells in vitro and inhibit SAS cell xenografted tumor in vivo. Toxicol Appl Pharmacol 382:114734 31470033 10.1016/j.taap.2019.114734

[CR15] Lai Y, Jiang J, Zhang H, Gong K (2024) Bibliometric Analysis of Curcumin Based on CiteSpace: Landscapes, Hotspots, and Frontiers. Drug design. Dev therapy 18:5743–5758 10.2147/DDDT.S494758 PMC11630724 39659947

[CR50] Lee AY, Fan CC, Chen YA, Cheng CW, Sung YJ, Hsu CP et al (2015) Curcumin Inhibits Invasiveness and Epithelial-Mesenchymal Transition in Oral Squamous Cell Carcinoma Through Reducing Matrix Metalloproteinase 2, 9 and Modulating p53-E-Cadherin Pathway. Integr cancer Ther 14(5):484–490 26036622 10.1177/1534735415588930

[CR55] Lee HM, Patel V, Shyur LF, Lee WL (2016) Copper supplementation amplifies the anti-tumor effect of curcumin in oral cancer cells. Phytomedicine: Int J phytotherapy phytopharmacology 23(12):1535–1544 10.1016/j.phymed.2016.09.005 27765374

[CR69] Lee GJ, Lim H, Seo JY, Kang KR, Kim DK, You JS et al (2022) Demethoxycurcumin induces apoptosis via inhibition of NF-κB pathway in FaDu human head and neck squamous cell carcinoma. Transl Cancer Res 11(5):1064–1075 35706794 10.21037/tcr-21-2410 PMC9189212

[CR80] Li L, Zhang X, Pi C, Yang H, Zheng X, Zhao L et al (2020) Review of curcumin physicochemical targeting delivery system. Int J Nanomed 9799–9821 10.2147/IJN.S276201 PMC7732757 33324053

[CR25] Li P, Pu S, Lin C, He L, Zhao H, Yang C et al (2022) Curcumin selectively induces colon cancer cell apoptosis and S cell cycle arrest by regulates Rb/E2F/p53 pathway. J Mol Struct 1263:133180

[CR52] Liao S, Xia J, Chen Z, Zhang S, Ahmad A, Miele L et al (2011) Inhibitory effect of curcumin on oral carcinoma CAL-27 cells via suppression of Notch-1 and NF-κB signaling pathways. J Cell Biochem 112(4):1055–1065 21308734 10.1002/jcb.23019

[CR121] Lin C, Tu C, Ma Y, Ye P, Shao X, Yang Z et al (2017) Curcumin analog EF24 induces apoptosis and downregulates the mitogen activated protein kinase/extracellular signal-regulated signaling pathway in oral squamous cell carcinoma. Mol Med Rep 16(4):4927–4933 28791378 10.3892/mmr.2017.7189

[CR1] Lissoni A, Agliardi E, Peri A, Marchioni R, Abati S (2020) Oral microbiome and mucosal trauma as risk factors for oral cancer: beyond alcohol and tobacco. A literature review. J Biol Regul Homeost Agents 34(6 Suppl 3):11–18 33386052

[CR12] Liu C, Yang X, Wu W, Long Z, Xiao H, Luo F et al (2018) Elaboration of curcumin-loaded rice bran albumin nanoparticles formulation with increased in vitro bioactivity and in vivo bioavailability. Food Hydrocolloids 77:834–842

[CR57] Liu T, Long T, Li H (2021) Curcumin suppresses the proliferation of oral squamous cell carcinoma through a specificity protein 1/nuclear factor-κB-dependent pathway. Exp Ther Med 21(3):202 33500696 10.3892/etm.2021.9635 PMC7818548

[CR76] Lopresti AL (2018) The Problem of Curcumin and Its Bioavailability: Could Its Gastrointestinal Influence Contribute to Its Overall Health-Enhancing Effects? Adv Nutr 9(1):41–50 29438458 10.1093/advances/nmx011 PMC6333932

[CR120] Lu YT, Lin CW, Su SC, Ho YT, Yeh FL, Hsin CH et al (2024) L48H37, a curcumin analog, suppresses matrix metalloproteinase-9 expression and activity to hamper nasopharyngeal cancer cell migration. Oral Oncol 159:107038 39284263 10.1016/j.oraloncology.2024.107038

[CR54] Ludwig N, Feldmann C, Spoerl S, Yerneni SS (2026) Extracellular vesicle-mediated delivery of curcumin suppresses tumor progression in murine oral squamous cell carcinoma. Cancers 18(10) 10.3390/cancers18101586 PMC13204880 42192945

[CR122] Ma T, Wang X, Wang Y, Hao Y, Yang X, Yan X et al (2024) Curcumin analogue AC17-loaded dissolvable microneedles activate FOXO3 and enhance localized drug delivery for oral squamous cell carcinoma treatment. Int J Pharm 661:124385 38925237 10.1016/j.ijpharm.2024.124385

[CR71] Manhas D, Dhiman S, Kour H, Kour D, Sharma K, Wazir P et al (2024) ADME/PK insights of crocetin: a molecule having an unusual chemical structure with druglike features. ACS omega 9(19):21494–21509 38764638 10.1021/acsomega.4c02116 PMC11097163

[CR59] Maulina T, Widayanti R, Hardianto A, Sjamsudin E, Pontjo B, Yusuf HY (2019) The Usage of Curcumin as Chemopreventive Agent for Oral Squamous Cell Carcinoma: An Experimental Study on Sprague-Dawley Rat. Integr Cancer Ther 18:1534735418822094 30616418 10.1177/1534735418822094 PMC6432668

[CR87] Mazzarino L, Loch-Neckel G, Bubniak Ldos S, Mazzucco S, Santos-Silva MC, Borsali R et al (2015) Curcumin-Loaded Chitosan-Coated Nanoparticles as a New Approach for the Local Treatment of Oral Cavity Cancer. J Nanosci Nanotechnol 15(1):781–791 26328442 10.1166/jnn.2015.9189

[CR33] Memarzia A, Khazdair MR, Behrouz S, Gholamnezhad Z, Jafarnezhad M, Saadat S et al (2021) Experimental and clinical reports on anti-inflammatory, antioxidant, and immunomodulatory effects of Curcuma longa and curcumin, an updated and comprehensive review. BioFactors 47(3):311–350 33606322 10.1002/biof.1716

[CR20] Menon VP, Sudheer AR (2007) Antioxidant and anti-inflammatory properties of curcumin. Adv Exp Med Biol 595:105–125 17569207 10.1007/978-0-387-46401-5_3

[CR7] Miller HI (2001) The story of Taxol: nature and politics in the pursuit of an anti-cancer drug. Nat Med 7(2):148

[CR38] Mirzaei H, Bagheri H, Ghasemi F, Khoi JM, Pourhanifeh MH, Heyden YV et al (2021) Anti-cancer activity of curcumin on multiple myeloma. Anticancer Agents Med Chem 21(5):575–586 10.2174/1871520620666200918113625 32951583

[CR41] Mishra A, Kumar R, Tyagi A, Kohaar I, Hedau S, Bharti AC et al (2015) Curcumin modulates cellular AP-1, NF-kB, and HPV16 E6 proteins in oral cancer. Ecancermedicalscience 9:525 25932049 10.3332/ecancer.2015.525 PMC4407748

[CR22] Moghadamtousi SZ, Kadir HA, Hassandarvish P, Tajik H, Abubakar S, Zandi K (2014) A review on antibacterial, antiviral, and antifungal activity of curcumin. Biomed Res Int 2014:186864 24877064 10.1155/2014/186864 PMC4022204

[CR62] Mohankumar K, Francis AP, Pajaniradje S, Rajagopalan R (2021) Synthetic curcumin analog: inhibiting the invasion, angiogenesis, and metastasis in human laryngeal carcinoma cells via NF-kB pathway. Mol Biol Rep 48(8):6065–6074 34355287 10.1007/s11033-021-06610-8

[CR3] Mohideen K, Krithika C, Jeddy N, Balakrishnan T, Bharathi R, Sankari SL (2021) A Meta-analysis of Oral Squamous Cell Carcinoma in Young Adults with a Comparison to the Older Group Patients (2014–2019). Contemp Clin Dent 12(3):213–221 34759676 10.4103/ccd.ccd_466_20 PMC8525813

[CR47] Molla S, Hembram KC, Chatterjee S, Nayak D, Sethy C, Pradhan R et al (2020) PARP inhibitor Olaparib Enhances the Apoptotic Potentiality of Curcumin by Increasing the DNA Damage in Oral Cancer Cells through Inhibition of BER Cascade. Pathol Oncol research: POR 26(4):2091–2103 31768967 10.1007/s12253-019-00768-0

[CR46] Molla S, Chatterjee S, Sethy C, Sinha S, Kundu CN (2021) Olaparib enhances curcumin-mediated apoptosis in oral cancer cells by inducing PARP trapping through modulation of BER and chromatin assembly. DNA Repair 105:103157 34144488 10.1016/j.dnarep.2021.103157

[CR99] Moravedeh R, Samadnezhad NZ, Asadalizadeh M, Abbasi M, Nadaki A (2025) Enhanced anticancer potential of curcumin-loaded liposomal nanoparticles in oral cancer treatment. Asian Pac J Cancer Biology 10(2):293–299

[CR92] Mukherjee D, Dash P, Ramadass B, Mangaraj M (2022) Nanocurcumin in Oral Squamous Cancer Cells and Its Efficacy as a Chemo-Adjuvant. Cureus 14(5):e24678 35663647 10.7759/cureus.24678 PMC9162890

[CR56] Neetha MC, Panchaksharappa MG, Pattabhiramasastry S, Shivaprasad NV, Venkatesh UG (2020) Chemopreventive Synergism between Green Tea Extract and Curcumin in Patients with Potentially Malignant Oral Disorders: A Double-blind, Randomized Preliminary Study. J Contemp Dent Pract 21(5):521–531 32690834

[CR77] Nelson KM, Dahlin JL, Bisson J, Graham J, Pauli GF, Walters MA (2017) The Essential Medicinal Chemistry of Curcumin. J Med Chem 60(5):1620–1637 28074653 10.1021/acs.jmedchem.6b00975 PMC5346970

[CR83] Nurohman I, Suhandi C, Chaerunisaa AY, Wilar G, Jafar G, Sriwidodo S (2026) Design evolution of curcumin-loaded nanostructured lipid carriers: formulation strategies, functional modifications, and mechanistic–translational perspectives. Int J Nanomed 586727 10.2147/IJN.S586727 PMC12988807 41836730

[CR49] Ohnishi Y, Sakamoto T, Zhengguang L, Yasui H, Hamada H, Kubo H et al (2020) Curcumin inhibits epithelial-mesenchymal transition in oral cancer cells via c-Met blockade. Oncol Lett 19(6):4177–4182 32391111 10.3892/ol.2020.11523 PMC7204627

[CR81] Pan-On S, Dilokthornsakul P, Tiyaboonchai W (2022) Trends in advanced oral drug delivery system for curcumin: A systematic review. J Controlled Release 348:335–345 10.1016/j.jconrel.2022.05.048 35654170

[CR31] Pourhanifeh MH, Mottaghi R, Razavi ZS, Shafiee A, Hajighadimi S, Mirzaei H (2021) Therapeutic applications of curcumin and its novel formulations in the treatment of bladder cancer: a review of current evidence. Anti-cancer Agents Med Chem 21(5):587–596 10.2174/1871520620666200807223832 32767956

[CR36] Pourhanifeh MH, Mottaghi R, Razavi ZS, Shafiee A, Hajighadimi S, Mirzaei H (2021) Therapeutic applications of curcumin and its novel formulations in the treatment of bladder cancer: a review of current evidence. Anticancer Agents Med Chem 21(5):587–596 10.2174/1871520620666200807223832 32767956

[CR17] Priyadarsini KI (2014) The Chemistry of Curcumin: From Extraction to Therapeutic Agent. Molecules 19(12):20091–20112 25470276 10.3390/molecules191220091 PMC6270789

[CR123] Raouf N, Darwish ZE, Ramadan O, Barakat HS, Elbanna SA, Essawy MM (2024) The anticancer potential of tetrahydrocurcumin-phytosomes against oral carcinoma progression. BMC Oral Health 24(1):1126 39327561 10.1186/s12903-024-04856-9 PMC11430579

[CR95] Rezaei F, Fesharakinia T, Gavanaroudi SB, Rezaeianjam M, Goodarzi MK, Abdollahi M et al (2024) Utilizing Niosome nanoparticles for the combined treatment of curcumin and cisplatin in oral cancer. Asian Pac J Cancer Biology 9(4):569–577

[CR117] Rodrigues FC, Kumar NA, Thakur G (2021) The potency of heterocyclic curcumin analogues: An evidence-based review. Pharmacol Res 166:105489 33588007 10.1016/j.phrs.2021.105489

[CR94] Saberian E, Jenčová J, Jenča A, Jenča A, Petrášová A, Jenča J et al (2025) Combination Therapy of Curcumin and Cisplatin Encapsulated in Niosome Nanoparticles for Enhanced Oral Cancer Treatment. Indian J Clin Biochem 40(1):59–66 39835233 10.1007/s12291-024-01279-9 PMC11741963

[CR37] Sadri Nahand J, Moghoofei M, Salmaninejad A, Bahmanpour Z, Karimzadeh M, Nasiri M et al (2020) Pathogenic role of exosomes and microRNAs in HPV-mediated inflammation and cervical cancer: a review. Int J Cancer 146(2):305–320 31566705 10.1002/ijc.32688 PMC6999596

[CR100] Saeidi N, Ansarikojouri M, Mardani M, Rezazadeh R, Goodarzi MK, Amiri F (2024) Enhancing the cytotoxic effects of Carboplatin and Cisplatin on liposomes in oral Cancer cells with Curcumin. Asian Pac J Cancer Biology 9(4):579–587

[CR30] Salehi M, Movahedpour A, Tayarani A, Shabaninejad Z, Pourhanifeh MH, Mortezapour E et al (2020) Therapeutic potentials of curcumin in the treatment of non-small-cell lung carcinoma. Phytother Res 34(10):2557–2576 32307773 10.1002/ptr.6704

[CR39] Salehi M, Movahedpour A, Tayarani A, Shabaninejad Z, Pourhanifeh MH, Mortezapour E et al (2020) Therapeutic potentials of curcumin in the treatment of non-small‐cell lung carcinoma. Phytother Res 34(10):2557–2576 32307773 10.1002/ptr.6704

[CR67] Semlali A, Contant C, Al-Otaibi B, Al-Jammaz I, Chandad F (2021) The curcumin analog (PAC) suppressed cell survival and induced apoptosis and autophagy in oral cancer cells. Sci Rep 11(1):11701 34083581 10.1038/s41598-021-90754-x PMC8175612

[CR103] Shen W, Qu Y, Jiang H, Wang H, Pan Y, Zhang Y et al (2022) Therapeutic effect and safety of curcumin in women with PCOS: A systematic review and meta-analysis. Front Endocrinol 13:1051111 10.3389/fendo.2022.1051111 PMC9646792 36387924

[CR42] Siddappa G, Kulsum S, Ravindra DR, Kumar VV, Raju N, Raghavan N et al (2017) Curcumin and metformin-mediated chemoprevention of oral cancer is associated with inhibition of cancer stem cells. Mol Carcinog 56(11):2446–2460 28618017 10.1002/mc.22692

[CR6] Siegel RL, Miller KD, Jemal A (2016) Cancer statistics, 2016. CA: a cancer journal for clinicians 66(1):7–30 10.3322/caac.21332 26742998

[CR90] Srivastava S, Mohammad S, Pant AB, Mishra PR, Pandey G, Gupta S et al (2018) Co-delivery of 5-Fluorouracil and Curcumin Nanohybrid Formulations for Improved Chemotherapy Against Oral Squamous Cell Carcinoma. J Maxillofac Oral Surg 17(4):597–610 30344406 10.1007/s12663-018-1126-z PMC6181858

[CR63] Su CW, Chuang CY, Chen YT, Yang WE, Pan YP, Lin CW et al (2021) FLLL32 Triggers Caspase-Mediated Apoptotic Cell Death in Human Oral Cancer Cells by Regulating the p38 Pathway. Int J Mol Sci 22:21 10.3390/ijms222111860 PMC8584525 34769290

[CR66] Su SC, Hsin CH, Lu YT, Chuang CY, Ho YT, Yeh FL et al (2023) EF-24, a Curcumin Analog, Inhibits Cancer Cell Invasion in Human Nasopharyngeal Carcinoma through Transcriptional Suppression of Matrix Metalloproteinase-9 Gene Expression. Cancers (Basel) 15(5) 10.3390/cancers15051552 PMC10000445 36900342

[CR34] Sung H, Ferlay J, Siegel RL, Laversanne M, Soerjomataram I, Jemal A et al (2021) Global Cancer Statistics 2020: GLOBOCAN Estimates of Incidence and Mortality Worldwide for 36 Cancers in 185 Countries. CA Cancer J Clin 71(3):209–249 33538338 10.3322/caac.21660

[CR79] Szczęsna W, Tsirigotis-Maniecka M, Lamch Ł, Szyk-Warszyńska L, Zboińska E, Warszyński P et al (2022) Multilayered curcumin-loaded hydrogel microcarriers with antimicrobial function. Molecules 27(4):1415 35209213 10.3390/molecules27041415 PMC8875356

[CR96] Takahashi M, Uechi S, Takara K, Asikin Y, Wada K (2009) Evaluation of an oral carrier system in rats: bioavailability and antioxidant properties of liposome-encapsulated curcumin. J Agric Food Chem 57(19):9141–9146 19757811 10.1021/jf9013923

[CR73] Tan Y, Wang Z, Xu M, Li B, Huang Z, Qin S et al (2023) Oral squamous cell carcinomas: state of the field and emerging directions. Int J Oral Sci 15(1):44 37736748 10.1038/s41368-023-00249-w PMC10517027

[CR14] Tonon CC, Panariello B, Chorilli M, Spolidorio DMP, Duarte S (2022) Effect of curcumin-loaded photoactivatable polymeric nanoparticle on peri-implantitis-related biofilm. Photodiagn Photodyn Ther 40:103150 10.1016/j.pdpdt.2022.103150 36244678

[CR70] Tsai SC, Yang JS, Lu CC, Tsai FJ, Chiu YJ, Kuo SC (2022) MTH-3 sensitizes oral cancer cells to cisplatin via regulating TFEB. J Pharm Pharmacol 74(9):1261–1273 35880728 10.1093/jpp/rgac056

[CR60] Utaipan T, Boonyanuphong P, Chuprajob T, Suksamrarn A, Chunglok W (2020) A trienone analog of curcumin, 1,7-bis(3-hydroxyphenyl)-1,4,6-heptatrien-3-one, possesses ROS- and caspase-mediated apoptosis in human oral squamous cell carcinoma cells in vitro. Appl Biol Chem 63(1):7

[CR84] Wahnou H, El Kebbaj R, Liagre B, Sol V, Limami Y, Duval RE (2025) Curcumin-based nanoparticles: advancements and challenges in tumor therapy. Pharmaceutics 17(1):114 39861761 10.3390/pharmaceutics17010114 PMC11768525

[CR82] Yadav KS, Soni G, Choudhary D, Khanduri A, Bhandari A, Joshi G (2023) Microemulsions for enhancing drug delivery of hydrophilic drugs: Exploring various routes of administration. Med Drug Discovery 20:100162

[CR27] Yang C, Zhu Q, Chen Y, Ji K, Li S, Wu Q et al (2024) Review of the protective mechanism of curcumin on cardiovascular disease. Drug Design, Development and Therapy, pp 165–192 10.2147/DDDT.S445555 PMC10838105 38312990

[CR72] Ye M, Huang W, Wu WW, Liu Y, Ye SN, Xu JH (2017) FM807, a curcumin analogue, shows potent antitumor effects in nasopharyngeal carcinoma cells by heat shock protein 90 inhibition. Oncotarget 8(9):15364–15376 28157708 10.18632/oncotarget.14970 PMC5362491

[CR119] Yin Y, Wang Z, Hu Y, Wang J, Wang YI, Lu Q (2024) Caffeic acid hinders the proliferation and migration through inhibition of IL-6 mediated JAK-STAT-3 signaling axis in human prostate cancer. Oncol Res 32(12):1881–1890 39574470 10.32604/or.2024.048007 PMC11576972

[CR24] Zahedi M, Salmani Izadi H, Arghidash F, Gumpricht E, Banach M, Sahebkar A (2023) The effect of curcumin on hypoxia in the tumour microenvironment as a regulatory factor in cancer. Archives Med science: AMS 19(6):1616–1629 10.5114/aoms/171122 PMC10696979 38058727

[CR102] Zeng L, Yang T, Yang K, Yu G, Li J, Xiang W et al (2022) Efficacy and safety of curcumin and curcuma longa extract in the treatment of arthritis: a systematic review and meta-analysis of randomized controlled trial. Front Immunol 13:891822 35935936 10.3389/fimmu.2022.891822 PMC9353077

[CR61] Zhang Z, Lin R, Liu Z, Yan T, Xia Y, Zhao L et al (2020) Curcumin analog, WZ37, promotes G2/M arrest and apoptosis of HNSCC cells through Akt/mTOR inhibition. Toxicol Vitro 65:104754 10.1016/j.tiv.2019.104754 31863822

[CR104] Zhang S, Wang J, Liu L, Sun X, Zhou Y, Chen S et al (2022) Efficacy and safety of curcumin in psoriasis: preclinical and clinical evidence and possible mechanisms. Front Pharmacol 13:903160 36120325 10.3389/fphar.2022.903160 PMC9477188

[CR78] Zhang J, Zhang Y, Wang H, Chen W, Lu A, Li H et al (2023) Solubilisation and Enhanced Oral Absorption of Curcumin Using a Natural Non-Nutritive Sweetener Mogroside V. Int J Nanomed 18:1031–1045 10.2147/IJN.S395266 PMC9968502 36855540

[CR75] Zhao C, Zhou X, Cao Z, Ye L, Cao Y, Pan J (2023) Curcumin and analogues against head and neck cancer: From drug delivery to molecular mechanisms. Phytomedicine 119:154986 37506572 10.1016/j.phymed.2023.154986

[CR58] Zhen L, Fan D, Yi X, Cao X, Chen D, Wang L (2014) Curcumin inhibits oral squamous cell carcinoma proliferation and invasion via EGFR signaling pathways. Int J Clin Exp Pathol 7(10):6438–6446 25400722 PMC4230161

